# Assessment of breast cancer and feasibility of subtyping of breast cancer using thoracoabdominal staging photon-counting detector computed tomography

**DOI:** 10.1038/s41598-026-58065-1

**Published:** 2026-06-22

**Authors:** Claudia Neubauer, Jakob Benedikt Weiß, Moisés F. Molina Fuentes, Oliver Gebler, Lisa Jung, Sarah Isabelle Huwer, Florin-Andrei Taran, Ingolf Juhasz-Böss, Fabian Bamberg, Marisa Windfuhr-Blum, Jakob Neubauer

**Affiliations:** 1 Department of Diagnostic and Interventional Radiology , University Medical Center Freiburg , Freiburg, Germany; 2 Department of Neuroradiology , University Hospital of Zurich , Zurich, Switzerland; 3 Department of Obstetrics & Gynecology , University Medical Center Freiburg , Freiburg, Germany; 4 Department of Gynecology and Gynecologic Oncology Center for Integrated Oncology Aachen Bonn Cologne Düsseldorf Medical Faculty and, University Clinic of Cologne, University of Cologne, Cologne, Germany

**Keywords:** Photon-counting detector computed tomography, Contrast-enhanced computed tomography, Breast cancer, Staging, Breast cancer subtyping, Cancer, Oncology

## Abstract

In this prospective study, we evaluated the performance of thoracoabdominal photon-counting detector computed tomography (PCD-CT) for breast cancer assessment with MRI as reference standard and iodine uptake as a potential marker for breast cancer subtyping. 75 women (mean age: 55.8 ± 13.9 years [SD]) with 79 newly diagnosed breast cancers and indication for staging CT received a prone-positioned contrast-enhanced thoracoabdominal PCD-CT and a breast MRI. Cancer visibility (median 1/1/1, IQR 0/1/1) and image quality (median 1/1/1, IQR 0/0/0) were rated excellent in PCD-CT on a 4-point Likert scale (1 = excellent, 4 = poor). Cancer size in PCD-CT correlated significantly with MRI (*p* < 0.001). Diagnostic accuracy was good for T-stage (pooled accuracy 0.814), focality (0.772), axillary (0.822) and internal mammary lymph nodes (0.981), moderate for ductal carcinoma in situ (0.591). A significantly lower maximum iodine uptake was revealed in cancer with ductal carcinoma in situ (*p* = 0.027), a significantly lower mean iodine uptake in triple negative cancers (*p* = 0.003). Thoracoabdominal PCD-CT demonstrated excellent cancer visibility with convincing results for assessing cancer size, T-stage, and lymph node status. Iodine uptake shows promising associations with triple negative breast cancer.

## Introduction

The local tumor stage of breast cancer dramatically impacts the therapeutic approach and provides crucial prognostic information^1^. Among imaging modalities, breast MRI possesses the highest sensitivity for detecting and assessing breast cancer. It outperforms clinical examination, ultrasonography, and mammography in estimating cancer size, identifying additional local disease, and detecting contralateral cancer spread^2–4^. This sensitivity applies for cases involving cancer associated or sole ductal carcinoma in situ (DCIS), which often manifest as non-mass enhancement^4,5^. In addition to breast MRI, dedicated contrast-enhanced cone-beam breast CT^6,7^ and dedicated photon-counting detector breast CT^8^ can be reliably utilized for breast diagnostics and cancer assessment. However, both breast MRI and dedicated breast CT come with additional time, costs, and the requirement for application of contrast agents. Furthermore, they are not available for all patients. In contrast to that, in our health care system and according to the national guidelines most patients newly diagnosed with breast cancer undergo a thoracoabdominal CT scan for breast cancer staging purposes which already includes a scan of both breasts and the axillary region.

Photon-counting detector computed tomography (PCD-CT) is an emerging technology with several advantages over dedicated breast CT. Unlike dedicated breast CT, thoracic PCD-CT can simultaneously examine and display both breasts, and additionally the thoracic wall and axillary region in the context of thoracoabdominal staging or other thoracic investigations. Due to direct conversion of photons into digital signals, the photon-counting detector technique allows for ultra-high spatial resolution, enhanced soft tissue contrast, increased iodine contrast, the ability to quantify iodine uptake, reduced artifacts, and lower radiation doses, making it a promising device for broad clinical applications^9–13^. In breast cancer staging, single phase contrast-enhanced thoracic PCD-CT in prone positioning has already demonstrated a higher diagnostic accuracy in assessing cancer size, T-classification, and detection of DCIS compared to digital mammography^14^. Another study using a three-phase thoracic PCD-CT protocol yielded promising results in evaluating breast cancer and regional lymphadenopathy^15^.

The aim of our study was to analyze the diagnostic performance of single-phase contrast-enhanced PCD-CT in prone position in the context of routine thoracoabdominal breast cancer staging compared with breast MRI, which served as the reference standard, to assess breast cancer. Our hypothesis was that thoracic PCD-CT performs comparably to breast MRI in local breast cancer assessment. Furthermore, we explored iodine uptake in breast cancer as a novel quantitative imaging marker and as a potential predictor for cancer biology.

## Results

### Study population

**Fig. 1:**
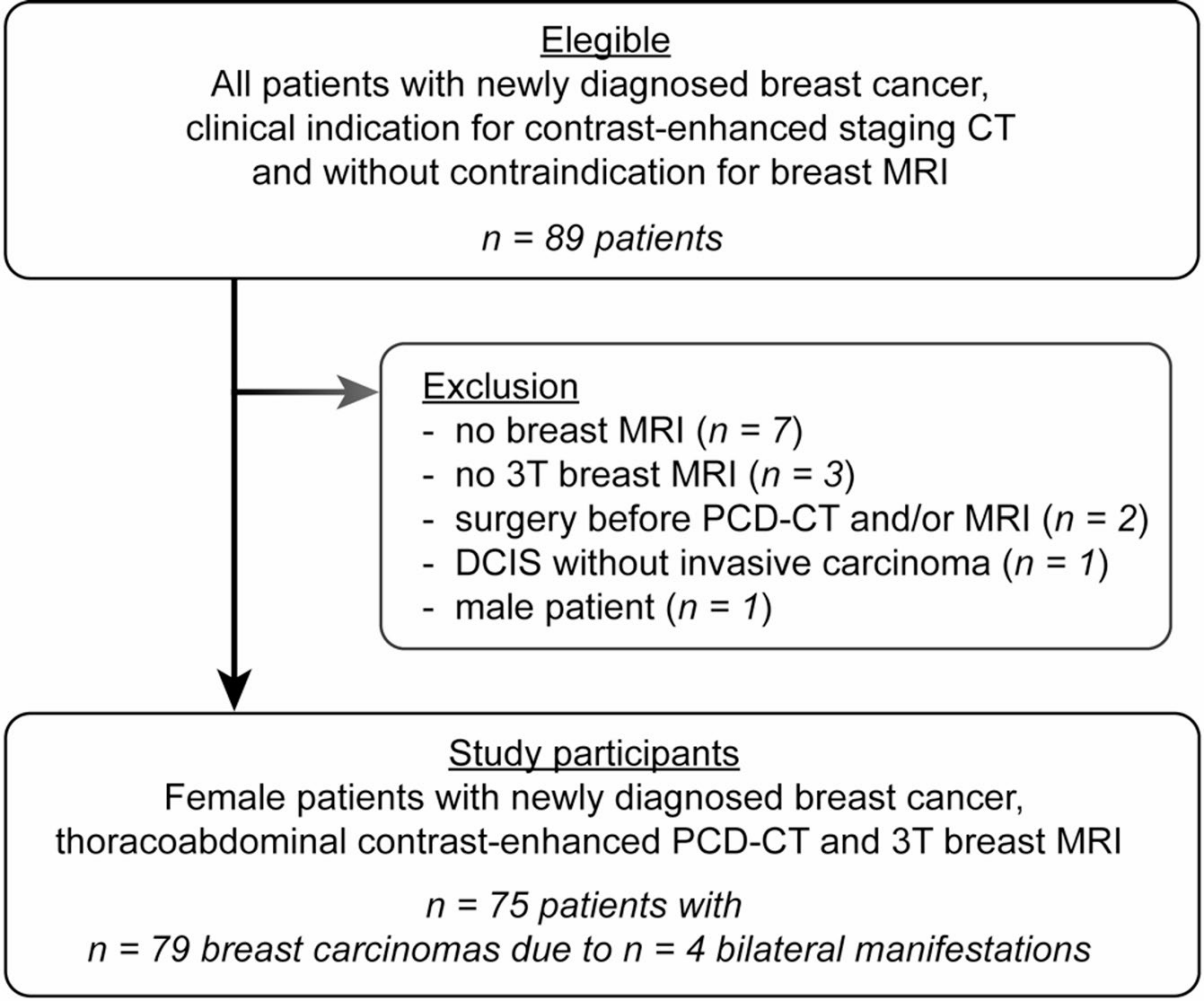
Flowchart for eligibility, exclusion and final collective of participants. DCIS ductal carcinoma in situ, PCD-CT photon-counting detector computed tomography, MRI magnetic resonance imaging.

Out of 89 eligible participants, 75 female participants (mean age: 55.8 years ± 13.9) with 79 breast cancers (bilateral cancer in 4 cases) were included in the study (Table 1). The exclusion criteria were incomplete imaging due to missing MRI (*n* = 7), examination performed with a 1.5 T MRI device (*n* = 3), prior breast surgery before PCD-CT and/or MRI (*n* = 2), histopathologically confirmed DCIS without invasive carcinoma (*n* = 1), or male sex (*n* = 1) (Fig. 1). Cancer characteristics such as cancer entity, TNM classification (TNM eighth edition), grading, receptor status, proliferation index (Ki67), and subsequent therapy were assessed for all participants (Table 2).

**Table 1:** Demographics of study participants.

	*n*
Number of enrolled female participants	75
Number of evaluated breast cancers	79
Unilateral left	32 (41% of cancers)
Unilateral right	39 (49%)
Bilateral	8 (10%)
Mean age ± standard deviation	55.8 ± 13.9
Menopausal status	
Premenopausal	26 (35% of women)
Postmenopausal	49 (65%)
Breast density as diagnosed in mammography	
A	8 (10%)
B	22 (28%)
C	32 (41%)
D	17 (22%)
Indication for thoracic CT	
Staging	75 (100%)

**Table 2:** Characteristics of assessed breast cancers.

Histopathology of breast cancer	n
No special type	41 (52%)
No special type + DCIS	33 (42%)
Lobular type	5 (6%)
Multifocality/Multicentricity of breast cancer per breast in histopathology	29 (37%)
> 1 cancer in biopsy	13 (45%)
> 1 cancer in surgical specimen	7 (24%)
> 1 cancer in biopsy and surgical specimen	3 (10%)
Large cancer infiltrating more than 1 breast quadrant with only 1 biopsy	6 (21%)
Local tumor stage, TNM	
T1	29 (37%)
T2	28 (35%)
T3	0
T4	22 (28%)
Regional lymph node status	
cN0	48 (61%)
cN1	25 (32%)
cN2	3 (4%)
cN3	3 (4%)
Distant metastasis, TNM	
cM0	68 (91%)
cM1 (3 oss, 1 hep, 1 oss/hep, 2 oss/hep/oth)	7 (9%)
Grading	
Grade 1	3 (4%)
Grade 2	52 (66%)
Grade 3	24 (30%)
ER	
Negative	15 (19%)
Positive	64 (81%)
PR	
Negative	24 (30%)
Positive	55 (70%)
HER2	
Negative	63 (80%)
Positive	16 (20%)
Proliferation index (Ki67)	
<10%	2 (3%)
>10%	77 (97%)
Neoadjuvant chemotherapy	
No	35 (44%)
Yes	40 (51%)
Palliative	4 (5%)

Total *n* = 79 tumors, except for metastasis total *n* = 75 patients.

### Breast cancer visibility, image quality, and confidence in diagnostic assessment in PCD-CT

**Fig. 2:**
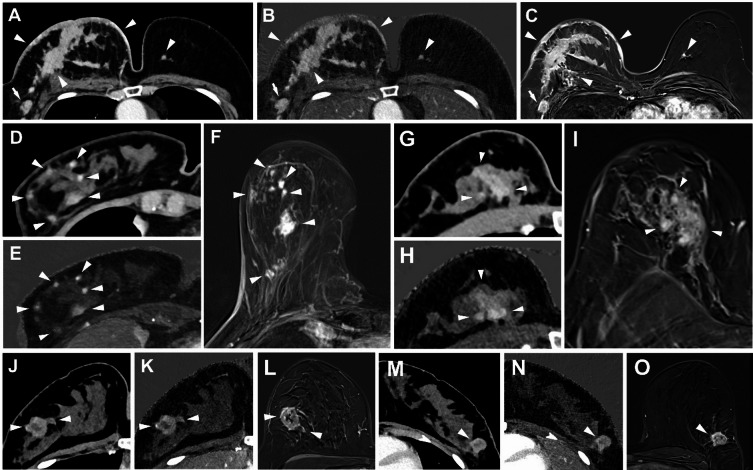
61-year-old participant with bilateral no special type right-sided Luminal B HER2 + G2 and left-sided Luminal B HER2- G1 breast cancer (arrowhead, **A**-**C**) and right-sided axillary lymph node metastasis (arrow) (A-C); 63-year-old participant with right-sided multifocal HER2 type G2 cancer (arrowheads, **D**-**F**), and 52-year-old participant with right-sided bifocal Luminal B HER2- G2 breast cancer (arrowheads, **G**-**I**), both of no special type associated with DCIS, and 32-year-old participant with bilateral unifocal triple negative G3 cancer with central necrosis (**J**-**O**). Shown are monoenergetic 65 keV reconstructions (A, D, G, J, M) and iodine maps (B, E, H, K, N) of contrast-enhanced thoracic photon-counting detector computed tomography, and subtraction images of contrast-enhanced MRI (C, F, I, L, O). Whilst the cancer of the first participant on the right side is clearly detectable but difficult to be measure accurately in all modalities due to diffuse expansion with dermal and pectoral muscle invasion (arrowheads), the left-sided cancer is clearly visible and exactly measurable despite its small size (arrowhead). In the second and third cases multifocality can clearly be seen but again cancer size is difficult to be accurately measured due to accompanying DCIS. In the last case (J-O), triple negative cancer is clearly visible and measurable in both breasts.

Cancer visibility was rated as very good in monoenergetic 65 keV reconstructions (median of 1/1/1 and IQR of 0/1/1 for rater 1/2/3, respectively), good to very good in the iodine map (median 1/1/2 and IQR 0/1/1.5), and moderate in the virtual non-contrast reconstruction (median 3/3/3 and IQR 2/2/2). Ratings for cancer visibility in monoenergetic 65 keV reconstructions and iodine maps with PCD-CT were non-inferior to ratings of cancer visibility obtained with the reference standard MRI (for each rater *p* < 0.001). The overall image quality of PCD-CT was rated as very good by all raters (median of 1/1/1, IQR 0/0/0). The overall confidence in diagnostic assessment was rated as very good with PCD-CT (median of 1/1/1, IQR 1/1/0), and non-inferior to confidence in diagnostic assessment with MRI (for each rater *p* < 0.001). Figure 2 provides a representative example of bilateral breast cancer and axillary lymph node metastasis.

### Breast cancer and lymph node assessment in PCD-CT

**Fig. 3:**
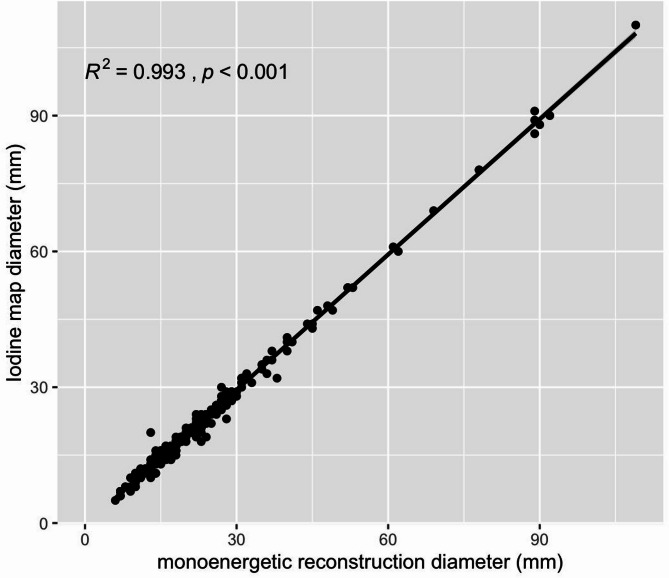
Correlation of measurements of largest cancer diameter in the monoenergetic 65 keV reconstructions (x-axis) and iodine map (y-axis) in thoracic contrast-enhanced photon-counting detector computed tomography.

**Fig. 4:**
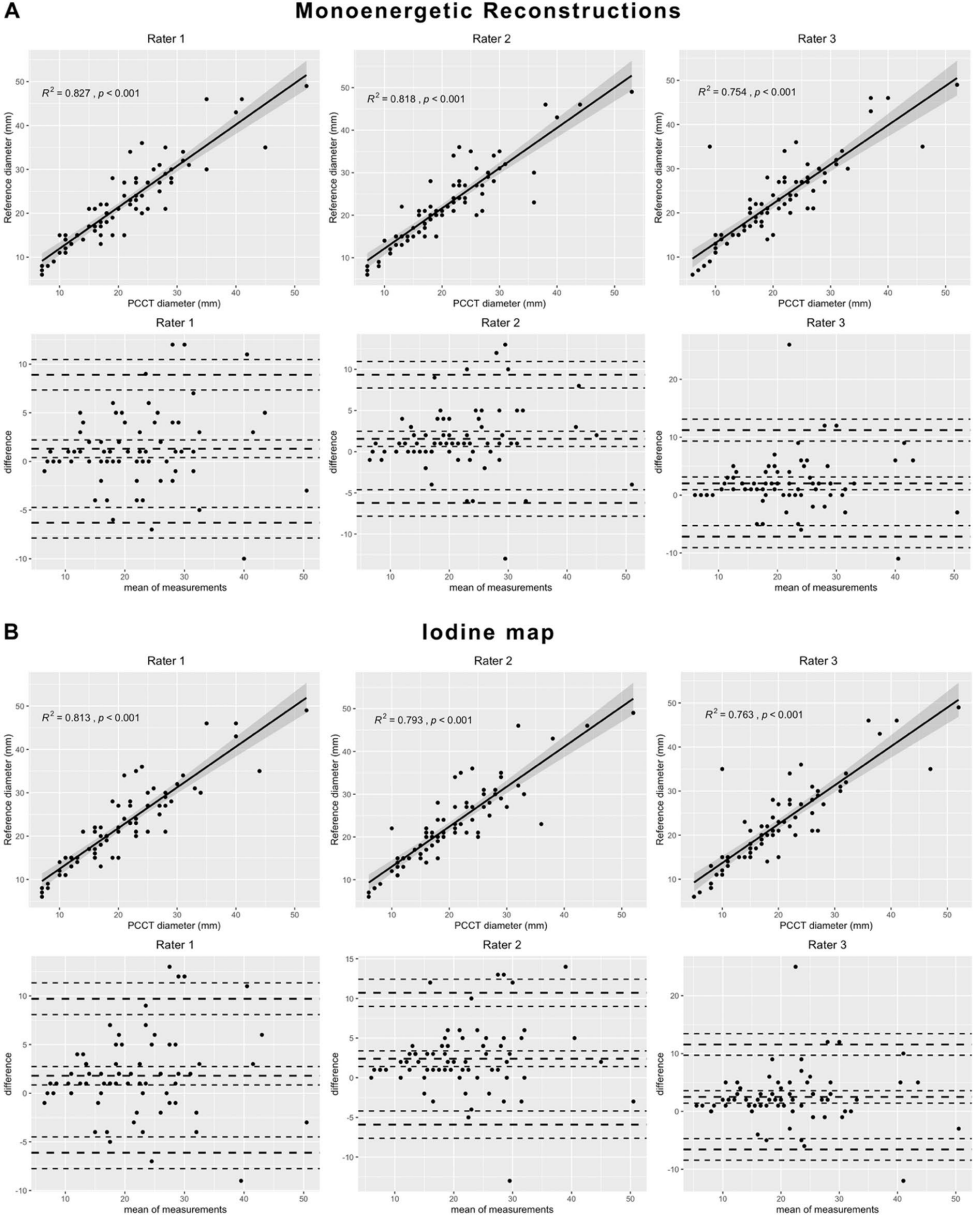
Correlation (scatter plots) and agreement (Bland-Altman plots) between cancer diameter measurements in monoenergetic 65 keV reconstructions (A, above) and the iodine map (B, below) in thoracic contrast-enhanced photon-counting detector computed tomography versus the reference diameter as measured in breast MRI for all raters.

Average correlation between all measurements of largest cancer diameter in monoenergetic 65 keV reconstructions and iodine maps in PCD-CT was excellent (Fig. 3). When comparing measurements, excluding diffuse and hardly measurable cancer expansion greater than 5 cm, with the reference standard MRI (*n* = 73), correlation and agreement were excellent for both monoenergetic 65 keV reconstructions (Fig. 4, upper panel) and iodine maps (Fig. 4, lower panel) for all raters.

The diagnostic accuracy for T-classification (TNM eighth edition) was altogether 0.814 (95% CI 0.759–0.862) with 0.873 (95% CI 0.780–0.938)/0.835 (95% CI 0.735–0.909)/0.734 (95% CI 0.623–0.827) for rater 1/2/3, for focality altogether 0.772 (95% CI 0.713–0.824) with 0.772 (95% CI 0.664–0.859)/0.798 (95% CI 0.692–0.880)/0.747 (95% CI 0.636–0.838) for rater 1/2/3. For suspicious axillary lymph nodes, the diagnostic accuracy was pooled 0.822 (95% CI 0.753–0.878) with 0.886 (95% CI 0.795–0.947)/0.760 (95% CI 0.650–0.849), and for internal mammary lymph nodes 0.981 (95% CI 0.946–0.996) with 0.975 (95% CI 0.912–0.997)/0.987 (95% CI 0.932–0.999) for rater 1/2. Sensitivity and specificity for depiction of suspicious lymph nodes in PCD-CT with reference to MRI was 0.885/0.769 and 0.887/0.755 for axillary lymph nodes and 0.833/1 and 0.986/0.986 (rater 1/2) for internal mammary lymph nodes.

Due to neoadjuvant chemotherapy, histopathological results for cancer extent were available in only 35 cases. When comparing the diagnostic assessment from all raters to those histopathological cancer stages, the diagnostic accuracy for T-stage assessment using PCD-CT was pooled 0.771 (95% CI 0.6793–0.8477) with 0.800 (95% CI0.631-0.916)/0.743 (95% CI 0.567–0.875)/0.771 (95% CI 0.599–0.896), a sensitivity of 0.840/0.800/0.800, and a specificity of 0.700/0.600/0.700 for rater 1/2/3. Therefore, in T-stage assessment PCD-CT performed similar to MRI, which showed an accuracy of 0.771 (95% CI 0.599–0.896), a sensitivity of 0.760, and a specificity of 0.800. Histopathological proof of multifocality or multicentricity via biopsy, surgery, both, or tumor extension was available in 29 cases. Those were detected by MRI with an accuracy of 0.946 (95% CI 0.867, 0.985), a perfect sensitivity of 1, and a specificity of 0.911. PCD-CT correctly detected multifocality with an accuracy of 0.784/0.824/0.757 (rater 1/2/3: 95% CI 0.673–0.871/0.718–0.903/0.643–0.849), a very good sensitivity 0.966/0.862/0.931, but a lower specificity of 0.667/0.800/0.644 compared to MRI. MRI and PCT-CT consistently identified clearly visible multifocality in 5 further cases for which definite histopathological confirmation was unavailable, owing to complete response or only residual in situ disease after neoadjuvant treatment. Comparison of evaluations of axillary lymph nodes to histopathological findings (from initial biopsy or primary surgery, *n* = 50 cases), yielded a diagnostic accuracy of 0.800 (95% CI 0.708–0.873), with a sensitivity of 0.759, and a specificity of 0.848 for PCD-CT, which was even better than MRI with an accuracy of 0.760 (95% CI 0.618–0.869), a lower sensitivity of 0.667, but a slightly higher specificity (0.870).

DCIS was depicted with a diagnostic accuracy of altogether 0.591 (95% CI 0.525–0.654) with 0.570 (95% CI 0.453–0.681)/0.658 (95% CI 0.543–0.761)/0.544 (95% CI 0.428–0.657) for rater 1/2/3 in comparison to MRI. When compared to histopathological findings, the diagnostic accuracy of DCIS was 0.633 (95% CI 0.517–0.739)/0.620 (95% CI 0.504–0.727)/0.506 (95% CI 0.391–0.621) with a for all raters pooled low sensitivity of 0.303 and a specificity of 0.652 for PCD-CT.

All or at least two raters overestimated tumors in PCD-CT compared with the MRI reference reading in 7.59% (6/79) of cases, including T4 stage assignment in five cases and classification of T2 instead of T1 tumors in one case. Tumors were underestimated in 8.86% (7/79) of cases, including failure to identify T4 tumors in four cases and classification of T1 instead of T2 tumors in three cases. The main reasons for discrepancies in T-stage assessment were differing interpretations of dermal infiltration (4 cases) and thoracic wall infiltration (5 cases).

When considering only the main tumor size for T classification without accounting for dermal or thoracic wall infiltration, all or at least two raters overestimated tumor size on PCD-CT, resulting in an upgraded T classification in 2.53% (2/79) of cases, and underestimated tumor size, resulting in a downgraded T classification in 11.39% (9/79) of cases compared with MRI. Overall, tumor size measurements on PCD-CT were slightly smaller than on MRI by 2.08/3.28/3.04 mm for raters 1/2/3, respectively (SD 5.23/7.92/10.05 mm). Thus, PCD-CT demonstrated a tendency toward slight tumor size underestimation compared with MRI. The largest relative measurement discrepancies (≥ 20%) occurred in tumors with a mean reference size of at least 25 mm.

In the subgroup with histopathological comparison, PCD-CT T-stage misclassification based on tumor size did not show the same tendency, with overestimation in four cases and underestimation in three cases. However, in four of the overestimated cases and in one underestimated case on PCD-CT, the MRI measurements were also incorrect.

Compared with histopathology, lymph node metastases were missed on PCD-CT in three cases despite histopathological confirmation, while metastases were suspected in five cases without histopathological evidence. In seven of these cases, the MRI reference assessment was also incorrect.

### Interrater reliability

Interrater reliability with intra-class correlation coefficient (ICC) showed good agreement for cancer size measurement in the monoenergetic 65 keV reconstructions (ICC = 0.841, *p* < 0.001) and the iodine map (0.841, *p* < 0.001), and a moderate agreement in the virtual non-contrast reconstruction (0.784, *p* < 0.001).

Interrater agreement was substantial for T-classification (0.707, *p* < 0.001), dermal infiltration (0.680, *p* < 0.001), focality (0.711, *p* < 0.001), suspicious axillary lymph nodes altogether as well as grouped according to the pathologic N classification with 0, 1–3, 4–9, > 10 lymph nodes (0.629, *p* < 0.001 and 0.634, *p* < 0.001), and for suspicious internal mammary lymph nodes (0.749, *p* < 0.001). Interrater agreement was moderate for number of lesions (0.545, *p* < 0.001), infiltration of pectoral muscles with 0.474 (*p* < 0.001), and only fair for the presence of DCIS suspicious findings (0.220, *p* < 0.001).

### Iodine uptake in breast cancer with molecular subtyping

**Fig. 5:**
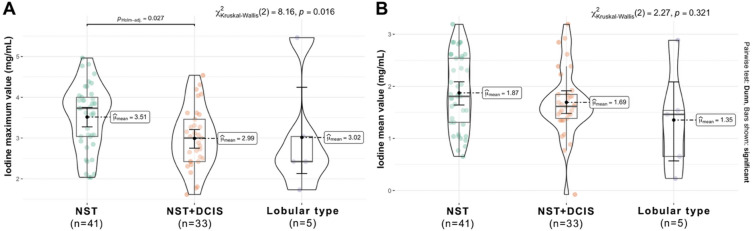
Measured maximum (**A**) and mean (**B**) iodine uptake in mg/mL for no special type (NST), no special type associated with DCIS (NST+DCIS), and the small subset of lobular type of breast cancer. A significant difference was shown for maximum iodine uptake (*p* = 0.016) between NST and NST+DCIS (*p* = 0.027) (A).

Enhancement, as quantified by iodine uptake using the iodine map, showed a significant difference between cancer entities. For measured maximum iodine uptake, the average and highest iodine values were 3.514 mg/mL (SD 0.79) and 4.961 mg/mL in NST cancers, 2.992 mg/mL (SD 0.74) and 4.538 mg/mL in NST cancers associated with DCIS, and 3.015 mg/mL (SD 1.44) and 5.461 mg/mL in the present small subset (*n* = 5) of lobular cancers (*p* = 0.016), which should therefore be interpreted with care. The post-hoc Dunn analysis revealed a significant difference between NST cancers and NST cancers with associated DCIS (*p* = 0.027) (Fig. 5A). However, no significant difference was observed in the measured mean iodine uptake with an average and highest value of 1.808 mg/mL (SD 0.69) and 3.192 mg/mL in NST cancers, of 1.763 mg/mL (SD 0.65) and 3.192 mg/mL in NST cancers with associated DCIS, and of 1.315 mg/mL (SD 1.02) and 2.885 mg/mL in lobular cancers (*p* = 0.321) (Fig. 5B).

**Fig. 6:**
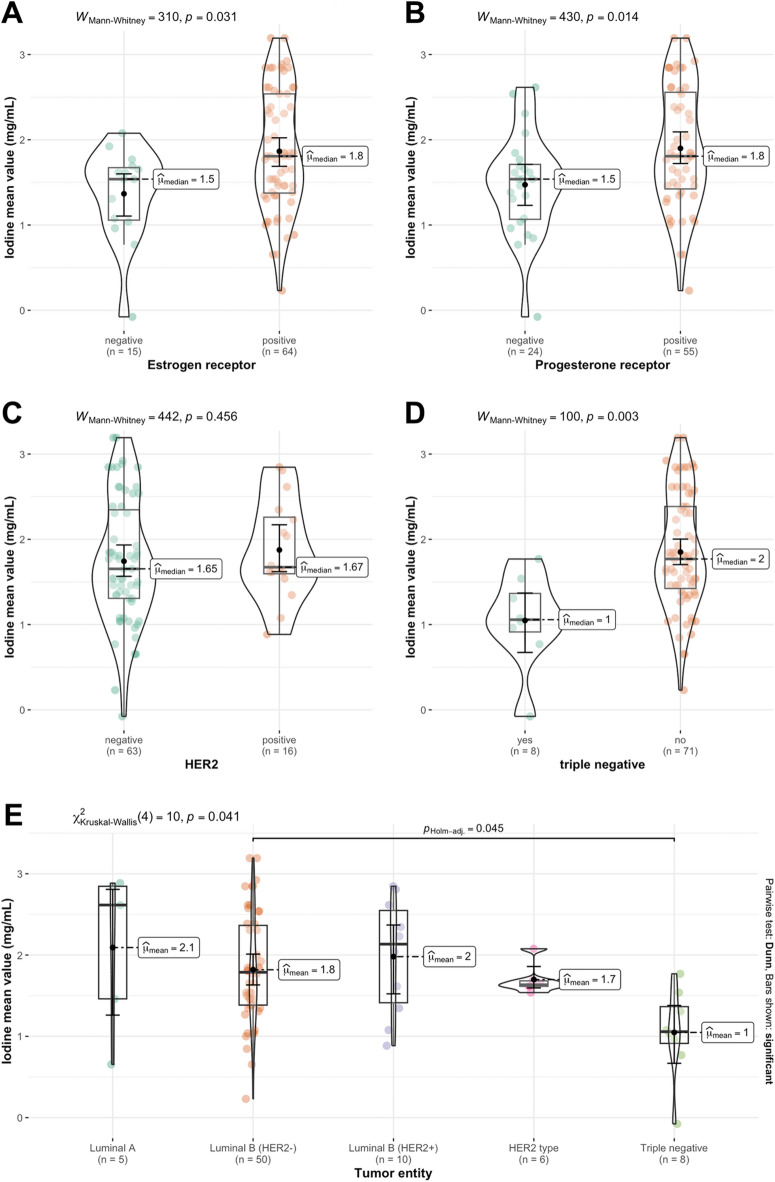
Mean iodine uptake in mg/mL for estrogen receptor status (**A**), progesterone receptor status (**B**), HER2 status (**C**), triple negative versus non-triple negative cancer (**D**), and all cancer entities (**E**). A significant difference was shown for mean iodine uptake with lower uptake in estrogen receptor negative and progesterone receptor negative breast cancer (*p* = 0.031 and 0.014) as well as in triple negative breast versus non-triple negative cancers (*p* = 0.003) or versus Luminal B (HER2-) (*p* = 0.045) cancer when differentiating all cancer types separately (*p* = 0.041) (E). No significant difference was observed in mean iodine uptake between HER2 negative and positive breast cancer (C). Corresponding figures for maximum iodine uptake are shown in the Figure, Supplemental Digital Content 1.

We found no significant difference in measured maximum iodine uptake regarding estrogen or progesterone receptor status and HER2 status (*p* = 0.975/0.424/0.386), between triple negative and all other cancer subtypes (*p* = 0.515), and altogether between all cancer subtypes (Luminal A, Luminal B HER2+, Luminal B HER2-, HER2 type and triple negative type) (*p* = 0.711, see Figure, Supplemental Digital Content 1, which illustrates these results). In contrast, in breast cancer with negative estrogen or progesterone receptor status, a significantly lower measured mean iodine uptake was noted compared to cancer with positive receptor status (*p* = 0.031 and *p* = 0.014, Fig. 6A/B). Although there was no significant difference in measured mean iodine uptake for HER2 status (*p* = 0.456, Fig. 6C), triple negative breast cancer demonstrated significantly lower measured mean iodine uptake compared to all other breast cancer subtypes, both collectively and separately (*p* = 0.003 and *p* = 0.041, Fig. 6D/E). Additionally, there was also no difference in measured maximum or mean iodine uptake across the three cancer grades (*p* = 0.714 and *p* = 0.237, see Figure, Supplemental Digital Content 2, which further illustrates these results).

The cancer-to-aorta ratios for measured maximum and mean HU measurements in the monoenergetic 65 keV reconstructions also showed no significant differences when comparing cancer types, grades, receptor status (in all cases *p* > 0.05).

Moreover, central necrosis was detected by MRI in 15/79 cancers and appeared to have a significant prevalence in triple negative cancer in both modalities, MRI (*p* = 0.005) and PCD-CT (*p* < 0.001/0.021/0.017 for rater 1/2/3) with an intermediate effect size (Cohen’s ω = 0.372 for MRI, 0.504/0.278/0.289 for PCD-CT).

## Discussion

In our study, we evaluated the diagnostic performance of thoracic contrast-enhanced PCD-CT in prone position, using breast MRI as the reference standard for breast cancer assessment. Cancer visibility and image quality were rated as very good and non-inferior to MRI (*p* < 0.001), with PCD-CT showing comparable performance to MRI for breast cancer size measurements (*p* < 0.001). Diagnostic accuracy was good for T-classification (0.814) and axillary lymph node assessment (0.822) and very good for internal mammary lymph node status (0.981). When compared to histopathological findings after surgery, PCD-CT demonstrated a good diagnostic accuracy for T-stage (0.771) and axillary lymph node assessment (0.800). However, the accuracy for detecting DCIS as non-mass enhancement was only moderate, both compared to MRI (0.591) and histopathology (0.633/0.620/0.506 for rater 1/2/3). Quantification of iodine uptake in breast carcinomas revealed a significant difference in maximum iodine uptake between NST cancer and NST cancer with associated DCIS (*p* = 0.027) as well as in mean iodine uptake for positive versus negative estrogen and progesterone receptor status (*p* = 0.031 and 0.014) and triple negative versus non-triple negative breast cancers (*p* = 0.003).

These findings align with previous studies that demonstrated a higher diagnostic accuracy for breast carcinoma size assessment and T-stage classification using thoracic PCD-CT in prone position compared to digital mammography^14^, as well as promising results in assessing breast cancer and regional lymphadenopathy using three-phase thoracic PCD-CT^15^. We opted for a one-phase protocol, as has been utilized in prior PCD-CT and dual-source CT studies^14,16–18^, and which demonstrated a high agreement between monoenergetic reconstructions and iodine maps for carcinoma measurements, consistent with prior results from dual energy CT^19^.

In terms of DCIS detection, previous studies reported a better performance of thoracic PCD-CT compared to digital mammography^14^, and a significant correlation between dedicated breast photon-counting CT and histopathology^20^. In our study, the diagnostic accuracy of PCD-CT for detecting DCIS as non-mass enhancement was only moderate compared to MRI (0.603) and histopathological findings (0.586), which may be attributed to generally lower enhancement and less defined spread of DCIS. Despite a particularly low sensitivity, the specificity of detecting DCIS was good (0.73), comparable to other studies using multidetector row CT^21–24^. We also observed a significantly lower mean iodine uptake in NST tumors that were accompanied by DCIS, which may possibly be attributed to cases of less defined transition of solid carcinoma into low enhancing DCIS. PCD-CT with its high spatial resolution^25^ may offer greater potential for assessing DCIS when additionally evaluating the detectability of suspicious microcalcifications as has already been explored in a breast phantom study for thoracic PCD-CT^26^ or in vivo for dedicated breast photon-counting detector CT in comparison with digital breast tomosynthesis^27^.

Also, the capability of differentiating between cancer subtypes such as triple negative and non-triple negative cancer based on iodine uptake as shown in this study aligns with previous research. Evaluations of contrast-enhanced chest CT have indicated a correlation between iodine content and hormone receptor expression^28^ as well as a capability to distinguish between different cancer types, particularly triple negative and non-triple negative cancer^29,30^. Furthermore, morphological tumor features have been shown to be helpful in differentiating breast cancer subtypes, receptor status, or aggressiveness with dedicated breast CT^31^ and breast MRI^32,33^. For example, triple negative cancer has been associated with rim enhancement^32,33^ or intratumoral heterogeneity in MRI^34^ or correspondingly with higher HU differences within the tumor in CT^31^.

## Limitations

Limitations of this study include its single-center design and the preselection of the cohort on the basis of an indication for thoracoabdominal staging CT, although this examination is routinely performed for initial staging of breast cancer in our healthcare system. In addition, our small cohort resulted in a limited and unbalanced sample size, including a small lobular carcinoma subgroup (*n* = 5), which precludes subgroup-specific conclusions. Furthermore, 42% of NST carcinomas were accompanied by DCIS, which may have influenced tumor size measurements and iodine uptake analyses. Iodine uptake segmentation was performed by a single rater only and without intra-observer variability assessment; consequently, interrater variability could not be assessed, representing an important methodological limitation for the molecular subtyping analyses. Accordingly, these findings should be regarded as hypothesis-generating and require validation in multirater studies. Lymph node assessment on PCD-CT was restricted to the two most experienced readers in senological and oncological radiology without a formal pre-specified justification, and no direct comparison with axillary ultrasound was performed, although axillary ultrasound remains the first-line modality for nodal staging and biopsy guidance in routine clinical practice. Another limitation is the prone acquisition geometry, while diagnostically advantageous for breast tissue visualization, it differs from the supine positioning routinely used for staging and follow-up CT. This inconsistency may complicate longitudinal dimensional comparisons and may affect assessment of the posterior pulmonary parenchyma relevant to M-stage evaluation. The interval between PCD-CT and breast MRI ranged from 0 to 17 days (mean 2.29 ± 6.1 days), which may introduce biologically meaningful changes between acquisitions, particularly in patients receiving neoadjuvant chemotherapy, although patients with longer intervals in this cohort did not undergo neoadjuvant treatment between the two examinations. Finally, histopathological reference data from surgical specimens were not available for all patients because a subset received neoadjuvant or palliative treatment; therefore, postsurgical histopathological comparisons were restricted to available cases, while MRI remained the primary imaging reference standard.

Altogether, from a clinical pathway perspective, thoracoabdominal PCD-CT in prone position should not be considered a replacement for dedicated breast MRI or axillary ultrasound. Breast MRI remains necessary for precise locoregional staging, particularly where DCIS extent or multifocality is decisive for surgical planning – a domain in which the moderate accuracy of PCD-CT is insufficient. Axillary ultrasound retains its irreplaceable role as the first-line modality for nodal staging and, critically, as the guide for core needle biopsy of suspicious lymph nodes as well as breast cancers in most cases, a function CT cannot substitute. The primary clinical value of this approach resides in its capacity to simultaneously assess both locoregional disease and distant metastases (M-stage) within a single acquisition already indicated for systemic staging, especially in cases where MRI is not available or contraindicated – a capability no dedicated breast modality provides.

In conclusion, in our study contrast-enhanced prone-positioned staging PCD-CT provided excellent cancer visibility and demonstrated a very good performance in comparison to MRI for assessing breast cancer size, T-stage, and lymph nodes. Iodine uptake measurements suggest that PCD-CT may be capable of distinguishing between cancer subtypes, such as NST cancer and NST cancer with associated DCIS and identifying triple negative breast cancer. Additionally, in contrast to other breast-specific imaging modalities, thoracic PCD-CT provides a comprehensive staging of breast cancer, including an assessment of the M-stage, all within a single examination.

Further studies are required to validate the efficacy and quality of thoracic PCD-CT in local breast cancer assessment and to explore its potential in cancer differentiation and molecular subtyping.

## Materials and methods

 This section is presented after the Discussion in line with the formatting requirements of the journal.

### Study design and population

This prospective study was conducted at a tertiary university medical center from December 2021 to August 2023. All participants with newly diagnosed biopsy-proven breast cancer, who had a current mammography, a medical indication for a thoracoabdominal contrast-enhanced CT in the context of breast cancer staging, and no contraindications for breast MRI were included after written informed consent. Pregnant or breast-feeding women were primarily not included in the study. Exclusion criteria were incomplete or no 3 T MRI imaging, imaging after surgery, no invasive cancer, or male patients (compare Fig. 1).

The present study, including the protocols, was approved by our Institutional Review Board (Ethics Committee of the University of Freiburg, number 21–1717) and in accordance with national guidelines and the Declaration of Helsinki. There are no conflicts of interest.

### Thoracic photon-counting detector CT examination and reconstructions

Dual source photon-counting detector CT (NAEOTOM-Alpha; Siemens Healthineers, Germany) examinations were performed in prone position with both breasts hanging freely between pillows comparable to positioning in breast MRI. Our protocol included a single-phase helical acquisition at 120 kVp and a quality reference of 142 mAs after bodyweight–adapted injection of iopromide (370 mg/mL; Bayer, Germany), followed by a saline chaser with a flow of 3 mL/s and a fixed delay of 85 s.

For evaluation of breasts and axillary lymph node status we added transversal reconstructions with a field of view equivalent to transversal breast MRI scans. These reconstructions were performed at a monoenergetic level of 65 keV, as an iodine map, and a virtual non-contrast reconstruction covering both breasts and the anterior thoracic wall including the axillary region with a fixed field of view of 34 cm, a matrix of 1024 × 1024 pixels, a slice thickness and increment of 2 mm, kernel Br40, and iterative reconstruction strength 3.

### Breast MRI examination

All participants underwent a multiparametric 3 T breast MRI (MAGNETOM Vida; Siemens Healthineers, Germany) within a period of 0 to 17 days (mean 2.29 days, ± 6.1 SD) following the PCD-CT examination. Breast MRI was performed with an 18-channel breast coil after application of 0.1 mmol/kg contrast agent (Gadoteridol, ProHance, Bracco, Konstanz, Germany). The MRI protocol consisted of a transversal T2 dixon vibe (including in-phase and water only image series), transversal diffusion-weighted imaging, always transversal native followed by four dynamic contrast-enhanced T1-weighted dixon vibe sequences every 90 s over 360 s altogether with generation of maximum intensity projections as well as subtracted images and additionally ultrafast dynamic breast imaging using TWIST sequences (missing in one case) during contrast agent application. All sequences were used for the evaluation of MRI as reference standard regarding imaging parameters.

### Image analysis and reference standard

The evaluation of pseudonymized PCD-CT examinations was performed by three different experienced radiologists (radiologist 1: 12 years CT imaging and 7 years breast imaging, radiologist 2: 10 years CT imaging and 6 months sole breast imaging, radiologist 3: 6 years CT imaging and 5 months sole breast imaging). Raters were informed about the side of breast malignancy. Otherwise, these raters were blinded to patient data.

The three raters independently evaluated cancer visibility in all reconstructions, overall confidence in diagnostic assessment, and overall image quality of PCD-CT and MRI imaging on a 4-point Likert scale (1 = excellent; 4 = poor). All raters measured the largest tumor diameter in all reconstructions, assessed tumor focality (unifocal versus multifocal/multicentric tumor), skin or pectoralis/thoracic wall infiltration, DCIS suspicious findings (non-mass enhancement). Additionally, axillary and internal mammary lymph nodes were assessed by the two most experienced raters in breast imaging and were rated as suspicious when they appeared with a marked asymmetry, a cortical thickening, loss of the fatty hilum, a round and enlarged shape, an irregular margin, a heterogeneous cortex, and/or surrounding edema.

Rater 1 also measured the mean and maximum Hounsfield Units (HU) of main tumors in monoenergetic 65 keV reconstructions and iodine maps using a ROI, that was created in the most representative axial slice with the maximum tumor diameter and by manually surrounding the whole solid tumor on that slice for analysis. In case of multifocality or multicentricity the main solid tumor was chosen for this segmentation. Another ROI was positioned in the ascending aorta to assess the relative contrast in cancer versus aortic contrast with mean and maximum HU in monoenergetic 65 keV reconstructions. From mean and maximum HU values in the iodine map, the iodine uptake value in mg/mL was calculated.

The reference standard (MRI) was evaluated by two experienced radiologists in consensus blinded to ratings from and independent from PCD-CT (radiologist with more than 25 years of CT and breast imaging and another radiologist with 14 years CT imaging and 7 years breast imaging). Regarding T-stage, focality, presence of DCIS and axillary lymph node assessment, histopathology was considered as reference standard in all available cases such as multiple biopsies or proof in specimens of multifocality, regarding T-stage and lymph nodes in cases without neoadjuvant treatment and primary surgery and/or prior lymph node biopsy via core needle biopsy to additionally assess sensitivity and specificity in those cases with definitive histopathology. Information about histologic cancer type and grade, hormone receptor and HER2 status, and Ki-67 index were provided in the histopathological reports after biopsy and/or surgery. All other clinical data were collected from the medical records.

### Statistics

For descriptive statistics continuous variables are reported as mean ± SD, whereas categorical variables are reported as counts and percentages. Likert-scale ratings of cancer visibility, confidence in diagnostic assessment and image quality are reported as median with interquartile range (IQR), and comparison with reference standard MRI was evaluated by paired non-inferiority tests, which are a variant of a t-test with exchanged hypotheses.

Correlations between cancer diameter measurements of PCD-CT reconstructions (monoenergetic 65 keV reconstruction versus iodine map) and with reference standard MRI were visualized by scatter plots and quantified by variance explained (adjusted R2) from linear regression. Agreement between PCD-CT and reference standard MRI was assessed by Bland-Altman plots.

Diagnostic accuracy was calculated for PCD-CT with MRI as the reference standard regarding correct T-stage classification, focality, and suspicious axillary and internal mammary lymph nodes. Additionally, in cases of histopathological assessment of lymph nodes, sensitivity and specificity were calculated for PCD-CT with histopathology as the reference standard for lymph node status. The same was performed for non-mass enhancement as a DCIS suspicious finding in case of histopathologically proven cancer associated DCIS or cancer surgery, for T-stage in case of primary surgery, or focality in case of histopathological proof.

Agreement between PCD-CT and MRI regarding cancer type (triple negative versus non-triple negative) and cancer necrosis was tested with Fisher’s or Chi-square test and Cohen’s ω for effect size.

Distribution of average and maximum cancer-to-aorta HU values and iodine uptake values for different histopathological cancer entities was visualized by violin plots and differences were evaluated using the Kruskal-Wallis test by ranks with post-hoc Dunn tests. Comparisons of HU values and iodine uptake for ER status, PR status, HER2 status, or between triple negative versus all other cancer entities together was performed with Mann-Whitney *U* test, between triple negative versus all other cancer entities separately with the Kruskal-Wallis test by ranks with post-hoc Dunn tests.

No formal correction for multiple testing was applied to the pre-specified iodine uptake analyses involving ER, PR, and HER2 status and derived subtype groupings. This decision was made because the number of comparisons was limited and correction was considered likely to increase the risk of type II error given the sample size and the partial intercorrelation among these biomarkers. Therefore, these analyses should be interpreted as exploratory.Interrater reliability was calculated using Fleiss’ kappa for nominal values and intra-class correlation with a two-way-random effects model and a single-rater unit for cancer size measurements. For Fleiss’ kappa *κ*, cutoffs of 0.2, 0.4, 0.6, 0.8, and 1 were considered to denote light, fair, moderate, substantial, and almost perfect agreement, respectively^35^. For intra-class correlation a value less than 0.5 indicated poor reliability, values between 0.5 and 0.75 moderate, between 0.75 and 0.9 good, and greater than 0.90 excellent reliability^36^.

P values < 0.05 were considered to indicate statistical significance. We used R version 4.3.2 and 4.5.0 for statistical analysis.

## Data Availability

Data generated or analyzed during the study are available from the corresponding author by request.

## Abbreviations

DCIS: Ductal carcinoma in situ
ER: Estrogen receptor
HER2: Human epidermal growth factor receptor 2
IQR: Interquartile range
NST: Breast cancer of no special type
PCD-CT: Photon-counting detector computed tomography
PR: Progesterone receptor
